# Supplementary material for: Atrial Fibrillation’s Influence on Short Sleep Duration Increases the Risk of Fatness in Management Executives
Source: Int J Environ Res Public Health. 2022 Apr 29;19(9):5438. doi: 10.3390/ijerph19095438 (PMC9103176; doi:10.3390/ijerph19095438)
Supplement: Supplementary file 1 [file ijerph-19-05438-s001.zip › ijerph-1668582-supplementary.pdf]

**Table S1. Sensitivity analysis for adjusted odds ratios (95% CI) between sleep duration and obesity (Male).**

| Variable | Sleep duration | | | |
|---|---|---|---|---|
| | **Very short (< 6 h)** | **Short (6-7 h)** | **Recommended (7-8h)** | **Long (>8 h)** |
| Total | *n* = 3,588 | *n* = 13,466 | *n* = 1,938 | *n* = 108 |
| Male (n=19,100) | 3.548(2.465-5.346) | 1.291(0.991-1.681) | Reference | 1.430(0.561-3.647) |

**Table S2. Sensitivity analysis for adjusted odds ratios (95% CI) between sleep duration and obesity (Female).**

| Variable | Sleep duration | | | |
|---|---|---|---|---|
| | **Very short (< 6 h)** | **Short (6-7 h)** | **Recommended (7-8h)** | **Long (>8 h)** |
| Total | *n* = 1292 | *n* = 4,677 | *n* = 826 | *n* = 58 |
| Female (n=6,853) | 6.342(4.769-8.759) | 1.644(0.809-3.340) | Reference | 2.845(0.588-13.760) |
